# Heat-stress-induced DNA methylation remodeling in cattle: a systematic evidence synthesis and meta-analytic feasibility assessment

**DOI:** 10.1016/j.vas.2026.100790

**Published:** 2026-07-29

**Authors:** Safa Bejaoui, Ikram Bensouf, Lotfi Mhamdi, Naceur M'Hamdi

**Affiliations:** a Research Unit of Biodiversity and Resource Development in Mountain Areas of Tunisia, LR13AGR02, Higher School of Agriculture of Mateur, 7030 Mateur, University of Carthage, Tunisia; b Laboratory of Animal, Genetic and Feed Resources (LRGAA), National Agronomic Institute of Tunisia, 43 Avenue Charles Nicolle, Tunis 1082, University of Carthage, Tunisia; c University Marie et Louis Pasteur, UMR Right, EPSI, College of Medicine Besançon, 19 Rue Ambroise Paré, 25030, France; d Monastir Biotechnology Institute, Taher Haddad Street, 5000 Monastir, Tunisia; e Animal, Genetic and Food Resources Laboratory (LRGAA), National Agronomic Institute of Tunisia, Carthage University, Tunis 1082, Tunisia

**Keywords:** Heat stress, DNA methylation, DNA hydroxymethylation, Epigenetics, Meta-analysis

## Abstract

Heat stress is an increasingly important constraint on cattle welfare, fertility, productivity, and developmental resilience. DNA methylation may mediate part of the molecular response to thermal exposure, but current studies differ widely in tissue type, exposure model, assay platform, and endpoint definition. This review systematically synthesizes evidence linking heat stress to DNA methylation changes in cattle and evaluates whether a quantitative meta-analysis of global methylation responses is currently feasible. A PRISMA-informed search was conducted for peer-reviewed bovine studies published between 2015 and early 2026. The PECO framework defined cattle or cattle-derived biological material as the population, heat stress or thermal challenge as the exposure, thermoneutral, cooled, or non-stressed conditions as comparators, and DNA methylation-related measures as outcomes. The predefined primary quantitative endpoint was global DNA methylation or hydroxymethylation reported as a continuous outcome. Locus-specific, promoter-level, DMC, DMR, and multi-omics studies were retained for structured qualitative synthesis. Global methylation studies often reported non-significant bulk changes, whereas sequencing-based and promoter-level studies revealed extensive tissue-specific and locus-specific methylation signatures involving oxidative stress, immune regulation, metabolism, endocrine signaling, and developmental programming. A formal pooled meta-analysis was not statistically defensible because fewer than three independent studies reported complete and comparable group-level data for the predefined global methylation endpoint. Heat stress in cattle appears to induce targeted, tissue-specific methylome remodeling rather than a consistent genome-wide shift in global methylation. Future studies should report extractable group-level methylation statistics, standardized heat-load metrics, tissue and cell-composition information, and phenotypic outcomes to enable robust meta-analysis. Beyond reporting standardization, future trial and cohort designs should prospectively incorporate paired global and locus-specific methylation endpoints to enable meta-analytic pooling within the next research cycle.

## Highlights

• Heat stress in cattle is associated with methylome remodeling across reproductive, immune, hepatic, blood, and developmental contexts.
• Available global 5mC/5hmC studies do not support a single universal direction of heat-induced methylation change.
• Locus-specific methylation signals repeatedly involve stress-response, immune, metabolic, endocrine, mitochondrial, and developmental pathways.
• A pooled effect size is currently inappropriate because endpoint definitions and extractable group-level statistics remain insufficient.
• Standardized heat-load metrics, transparent methylation statistics, open matrices, and phenotype-linked designs are essential for future high-confidence meta-analysis.

**Implications**

This paper discusses the impact of heat stress on the regulation of DNA in cattle. The increasing temperatures worldwide have led to negative impacts such as decreased welfare, fertility, and productivity in animals, which has implications for losses incurred by farmers. It is established that heat stress does not affect all the DNA in an organism, but only particular genes involved in processes such as immunity, metabolism, and development. This paper identifies the importance of developing better techniques to study heat stress impacts on livestock.

Alt-text: Unlabelled box dummy alt text

## 1 Introduction

Heat stress has become a central biological, welfare, and sustainability challenge in cattle production. Rising ambient temperature, more frequent heat waves, and high relative humidity increase the thermal load imposed on animals, especially high-producing dairy cows whose metabolic heat production is already elevated. Heat stress reduces dry-matter intake, disrupts metabolic and endocrine homeostasis, alters immune function, increases respiratory and cardiovascular strain, depresses milk yield, and compromises reproductive performance (Polsky and von Keyserlingk, 2017; Lacetera, 2019; Roth, 2020; Rhoads, 2023). These effects are not marginal production losses; they affect animal welfare, farm profitability, disease susceptibility, and the long-term sustainability of cattle systems under climate change. Heat stress can impair follicular dynamics, oocyte maturation, fertilization, early embryonic development, uterine function, and pregnancy maintenance. Importantly, the biological consequences are not limited to the directly exposed animal. Maternal heat load during gestation can influence calf growth, immunity, metabolism, thermoregulation, and later-life performance, indicating that thermal exposure can become biologically embedded during sensitive developmental windows (Skibiel et al., 2021; Laporta et al., 2024; Boucher et al., 2026). This intergenerational dimension makes bovine heat stress a priority topic for climate-resilient livestock science.

Epigenetic regulation provides a plausible mechanistic bridge between thermal exposure and altered phenotype. DNA methylation, most commonly cytosine methylation at CpG sites, contributes to chromatin organization, transcriptional regulation, genomic stability, and cellular identity. Unlike DNA sequence variation, methylation is responsive to environmental conditions, developmental stage, oxidative status, endocrine signaling, nutrition, inflammation, and tissue composition. Heat stress may therefore alter not only immediate physiology but also regulatory programs linked to immune competence, metabolism, reproduction, and developmental plasticity (Wang et al., 2021; Livernois et al., 2021; Del Corvo et al., 2021).

The emerging bovine literature suggests that the heat-stress methylation signal is complex. Studies measuring global DNA methylation or hydroxymethylation often report limited or statistically nonsignificant bulk changes, even when oocyte quality, gene expression, or cellular stress markers are altered (Diaz et al., 2021; Aalam et al., 2022). In contrast, genome-wide and promoter-level studies identify differentially methylated cytosines, regions, or promoters in pathways related to oxidative stress, immune regulation, apoptosis, metabolic adaptation, endocrine signaling, and developmental programming (Sajjanar et al., 2024; Laporta et al., 2025; Halli et al., 2025; Boucher et al., 2026). These observations are not contradictory. They indicate that heat stress may produce targeted regulatory remodeling without substantially changing the average methylation level across the genome.

Heat stress also induces oxidative stress, which provides an important mechanistic bridge between thermal load and epigenetic remodeling. Increased body temperature and altered nutrient partitioning can intensify mitochondrial reactive oxygen species (ROS) production, disturb antioxidant capacity, promote lipid peroxidation, and activate redox-sensitive pathways such as Nrf2/HO-1. In cattle, these processes are biologically relevant because oxidative imbalance can affect oocyte competence, immune-cell function, hepatic metabolism, inflammatory signaling, and embryo development. Oxidative stress can also interact with DNA methylation machinery by altering one-carbon metabolism, methyl-donor availability, TET-dependent hydroxymethylation, and stress-responsive transcriptional programs. Therefore, heat-induced methylation changes should be interpreted within a broader redox-stress framework rather than as isolated epigenetic events.

A scientifically defensible meta-analysis requires comparable outcomes. Global 5-methylcytosine or 5-hydroxymethylcytosine signal intensity, promoter-specific methylation, counts of differentially methylated cytosines, differentially methylated regions, pathway enrichment results, and multi-omics ordination metrics estimate different biological quantities. Pooling them solely because they are methylation-related would create a misleading summary effect. The present review therefore adopts a restricted meta-analytic framework: global DNA methylation or hydroxymethylation is treated as the predefined continuous primary endpoint, while locus-specific and multi-omics methylome studies are synthesized separately as mechanistic evidence.

Accordingly, this review aimed to identify bovine heat-stress methylation studies, classify endpoints according to quantitative poolability, assess the feasibility of a restricted global 5mC/5hmC meta-analysis, synthesize locus-specific methylome evidence across biological matrices, and define reporting priorities for future meta-analysis and biomarker development.

## 2 Materials and methods

This review was designed as a PRISMA-informed systematic evidence synthesis and meta-analytic feasibility assessment. A prospective protocol was not registered; this limitation is explicitly acknowledged. No pooled model was fitted because the prespecified minimum threshold for complete, comparable endpoint-A data was not met. The review, therefore, separates two questions that are often conflated: whether heat stress is associated with bovine methylome remodeling, and whether current reporting supports a statistically defensible pooled estimate of global methylation response. The review followed the main PRISMA elements applicable to a non-registered systematic evidence synthesis: a structured PECO question, explicit eligibility criteria, two-stage screening, endpoint hierarchy, transparent study-selection flow, and prespecified criteria for deciding whether a pooled model would be defensible. Deviations from full PRISMA compliance were the absence of prospective protocol registration and the use of a focused English-language targeted evidence map rather than an unrestricted all-language database harvest.

### 2.1 Review question and PECO framework

The main review question was: in cattle exposed to heat stress, what evidence is there that DNA methylation is altered compared with thermoneutral, cooled, or nonstressed controls? The primary quantitative question was narrower: among studies reporting global DNA methylation or hydroxymethylation as continuous outcomes, is there sufficient extractable information to estimate a pooled standardized mean difference between heat-stressed and control animals or cells?

Table 1 defines the methodological boundary of the study. The broad PECO question captures bovine methylation responses to heat stress, but the quantitative endpoint is deliberately restricted to global 5mC/5hmC values. This prevents promoter methylation, DMC/DMR counts, and multi-omics separation metrics from being treated as interchangeable effect sizes.

**Table 1:** PECO framework and endpoint hierarchy for the systematic review.

PECO element	Definition used in this review	Examples
Population	Cattle, including Bos taurus, Bos indicus, and crossbred animals; in vivo tissues, embryos, oocytes, and cultured bovine cells were eligible if derived from cattle.	Dairy cows, beef cows, calves, oocytes, embryos, PBMCs, blood, liver.
Exposure	Heat stress, thermal challenge, seasonal summer heat, high THI, in vitro heat shock, prenatal heat stress, or postnatal heat exposure.	Summer THI >72, late-gestation heat stress, PBMC heat shock at elevated temperature.
Comparator	Thermoneutral, cooled, spring/cool season, non-heat-shocked or management-control group.	Spring vs. summer; cooled dams vs. heat-stressed dams; control vs. heat-shocked cells.
Outcomes	Primary endpoint A: global DNA methylation or hydroxymethylation as continuous values. Secondary endpoints: promoter methylation, DMCs, DMRs, DMGs, pathway enrichment, and multi-omics methylation signatures.	5mC/5hmC intensity; RRBS/WGBS DMCs; methylated promoters; methylation-transcriptome integration.

### 2.2 Search strategy and study selection

Abbreviations used in the Methods and Results are as follows: PECO, Population-Exposure-Comparator-Outcome; PRISMA, Preferred Reporting Items for Systematic Reviews and Meta-Analyses; 5mC, 5-methylcytosine; 5hmC, 5-hydroxymethylcytosine; DMC, differentially methylated cytosine; DMR, differentially methylated region; DMG, differentially methylated gene; RRBS, reduced-representation bisulfite sequencing; WGBS, whole-genome bisulfite sequencing; PBMC, peripheral blood mononuclear cell; THI, temperature-humidity index; ROS, reactive oxygen species; SMD, standardized mean difference (Table 2).

**Table 2:** Database-specific search strategy, date restrictions, and language limitations.

Database/source	Complete search syntax used	Limits/notes
PubMed	(cattle OR bovine OR Bos taurus OR Bos indicus OR dairy cow) AND (heat stress OR thermal stress OR heat shock OR high temperature OR temperature-humidity index OR THI) AND (DNA methylation OR DNA hydroxymethylation OR methylome OR epigenetic)	English; peer-reviewed primary studies; 2015-31 Jan 2026.
Scopus	TITLE-ABS-KEY(cattle OR bovine OR "Bos taurus" OR "Bos indicus" OR "dairy cow") AND TITLE-ABS-KEY("heat stress" OR "thermal stress" OR "heat shock" OR "high temperature" OR "temperature-humidity index" OR THI) AND TITLE-ABS-KEY("DNA methylation" OR hydroxymethylation OR methylome OR epigenetic)	English; articles and reviews screened, but only primary bovine methylation studies retained as included studies; 2015-31 Jan 2026.
Web of Science	TS=(cattle OR bovine OR "Bos taurus" OR "Bos indicus" OR "dairy cow") AND TS=("heat stress" OR "thermal stress" OR "heat shock" OR "high temperature" OR "temperature-humidity index" OR THI) AND TS=("DNA methylation" OR hydroxymethylation OR methylome OR epigenetic)	English; Science Citation Index and related collections; 2015-31 Jan 2026.
Google Scholar / publisher websites	Targeted combinations: "bovine heat stress DNA methylation"; "cattle thermal stress methylome"; "dairy cow heat stress epigenetics"; "prenatal heat stress calf DNA methylation"; "oocyte heat stress DNA hydroxymethylation"; "heat stress PBMC promoter methylation cattle"	Complementary manual search; relevance-ranked results and publisher-first records were checked to avoid missing recent bovine studies.

Searches were conducted in PubMed, Scopus, Web of Science, Google Scholar, and publisher websites. The final update included records available from 1 January 2015 to 31 January 2026. Searches were limited to English-language, peer-reviewed primary studies for eligibility assessment; review articles were used only for contextual background. Search terms combined bovine/cattle terms, heat-stress terms, and methylation/epigenetic terms. Google Scholar and publisher-site searches were used as complementary manual searches to capture recently indexed or publisher-first records. Records were screened first by title/abstract and then by full text or publisher record. When numerical methylation values were unavailable in the main text, tables, figures, or supplementary material, the study remained eligible for qualitative synthesis but was not considered immediately poolable for endpoint A.

### 2.3 Inclusion and exclusion criteria

Studies were considered eligible when they were peer-reviewed experimental, observational, or mechanistic investigations conducted in cattle or in cattle-derived biological materials, including cells, tissues, oocytes, embryos, blood, PBMCs, or liver, and when they directly evaluated DNA methylation or DNA hydroxymethylation in relation to heat-stress exposure. Eligible designs included in vivo studies based on seasonal heat stress, controlled comparisons between heat-stressed and cooled or thermoneutral animals, prenatal or postnatal heat-stress models, and in vitro heat-shock experiments using bovine material. For the primary meta-analytic assessment, only studies reporting group-level global methylation or hydroxymethylation values with an appropriate comparator were considered quantitatively eligible. Studies that reported only differentially methylated cytosines, differentially methylated regions, promoter-specific methylation signatures, pathway enrichment results, or multi-omics clustering without extractable global methylation values were not included in the primary meta-analysis, but were retained for qualitative and mechanistic discussion because of their biological relevance. Studies were excluded entirely when they were conducted in non-bovine species, used non-thermal stress models, assessed epigenetic endpoints other than DNA methylation alone, lacked an appropriate control or comparator group, or consisted of opinion articles, narrative comments, or non-primary research reports.

### 2.4 Data extraction

For each eligible study, the following information was extracted: first author and year; biological material; breed or genotype; animal age or developmental stage; heat-stress context and metric; comparator; methylation assay; endpoint type; sample size; principal methylation result; availability of numerical data; and suitability for primary endpoint A. Where possible, numerical values were recorded as mean, standard deviation, or standard error, n per group, and p values. If only figures were available, the article was marked as potentially recoverable by digitization but not entered into a pooled model unless numerical extraction could be verified.

### 2.5 Risk-of-bias and evidence grading

Risk of bias was evaluated with an animal-epigenomics adaptation of standard observational and experimental criteria. Domains included clarity of heat-stress definition, adequacy of comparator, biological replication, assay validity, control for breed/parity/stage/cell composition, completeness of methylation reporting, and transparency of statistical analysis. Evidence certainty was not downgraded simply because a study used genome-wide endpoints, but such endpoints were not pooled with global methylation assays.

To strengthen academic rigor, the risk of bias was summarized at the study level using ordered judgments such as low, moderate, high, or unclear risk. The most relevant domains are heat-stress definition, comparator adequacy, biological replication, methylation-assay validity, control of breed, parity, developmental stage and cell composition, completeness of numerical reporting, and transparency of statistical analysis. This approach makes the quality assessment more auditable and avoids relying only on a narrative judgment.

### 2.6 Statistical analysis plan

The primary quantitative endpoint was defined as Hedges' g, a small-sample-corrected standardized mean difference in global DNA methylation or hydroxymethylation between heat-stressed and comparator groups. Negative values indicated lower methylation or hydroxymethylation under heat stress. The model was specified a priori because tissue type, breed, developmental stage, heat-stress model, exposure duration, and methylation assay were expected to introduce genuine biological and methodological heterogeneity. This technical plan is retained only to document the prespecified decision rule; no pooled model was fitted because the required minimum of three complete independent endpoint-A comparisons was not met.

The proposed random-effects model was g_i_ = θ + u_i_ + e_i_, where g_i_ represents the study-specific effect size, theta the overall mean effect, u_i_ the between-study random component, and e_i_ the within-study sampling error. The assumptions were u_i_ approximately N (0, tau2) and e_i_ approximately N (0, v_i_), with inverse-variance weights defined as w_i_ = 1/(v_i_ + tau2). Between-study variance would be estimated using DerSimonian-Laird for exploratory purposes, while the Hartung-Knapp-Sidik-Jonkman adjustment would be preferred for inference when the number of studies was small.

Importantly, this model was prespecified but not fitted as a pooled meta-analysis because the minimum pooling criterion was not met. A pooled estimate was planned only if at least three independent studies or study arms reported compatible endpoint-A data, including mean, dispersion estimate, and sample size for both heat-stressed and comparator groups. This rule was applied to avoid a statistically fragile forest plot based on sparse or non-comparable evidence.

Studies reporting only differentially methylated cytosines, differentially methylated regions, promoter methylation, pathway enrichment, or multi-omics clusters were excluded from the primary pooled model because these outcomes do not estimate the same biological quantity as global methylation intensity. They were retained for structured qualitative synthesis because they provide essential mechanistic information on tissue-specific and locus-specific methylome remodeling.

If future evidence becomes sufficient, subgroup analyses may be performed by biological matrix, including reproductive cells, blood or PBMCs, liver, embryos, prenatal exposure, and postnatal exposure. Meta-regression could also examine heat-stress intensity, exposure duration, breed type, developmental stage, and assay platform, but only when enough independent comparisons are available to avoid overfitting. Publication-bias tests would not be interpreted when fewer than ten studies are available.

## 3 Results and discussion

### 3.1 Study selection and evidence-map interpretation

The updated search and manual evidence review identified a focused but heterogeneous body of bovine heat-stress methylation studies. The final evidence map included studies in oocytes, early embryos, peripheral blood mononuclear cells, whole blood, and liver. Exposure models ranged from in vitro acute heat shock to seasonal in vivo heat stress, preweaning postnatal heat stress, and prenatal heat stress during late gestation (Diaz et al., 2021; Aalam et al., 2022; Laporta et al., 2025; Boucher et al., 2026). The most important methodological finding was endpoint divergence: only a minority of studies measured global methylation as a continuous outcome, whereas most recent studies used genome-wide or promoter-level methylome approaches (Livernois et al., 2021; Del Corvo et al., 2021; Sajjanar et al., 2024; Halli et al., 2025). Table 2 summarizes the study-selection flow and shows why the review reaches a restricted meta-analytic conclusion rather than a full pooled meta-analysis.

Table 3 quantifies the main methodological result of the review. Although 12 primary studies were suitable for systematic synthesis, fewer than three provided complete endpoint-A data. This means that the evidence base is mature enough to support a strong systematic review, but not yet mature enough for a definitive pooled effect size.

**Table 3:** PRISMA-style selection summary for the updated review.

Flow stage	Number	Interpretation
Records identified after update searches and manuscript revision	47	Represents the targeted evidence base rather than an exhaustive all-language database harvest.
Records excluded after title/abstract screening	28	Mostly non-bovine, non-heat-stress, or non-methylation studies.
Full-text or publisher-record articles assessed	19	Included experimental and observational bovine epigenomics studies plus relevant mechanistic papers.
Primary methylation studies included in systematic synthesis	12	Studies directly assessed DNA methylation or hydroxymethylation under heat-stress conditions.
Studies conceptually aligned with endpoint A	5	Measured global or global-like DNA methylation/hydroxymethylation.
Studies immediately quantitatively poolable numerical data	<3 with comp	Insufficient for a statistically defensible pooled meta-analysis.

The 47 records should be interpreted as a focused evidence map built around bovine heat-stress methylation terms, not as an unrestricted all-language bibliographic harvest. Among the endpoint-A studies, only one evidence stream provided immediately recoverable global 5mC/5hmC summary values suitable for illustration, whereas additional candidate studies required verified article-level extraction or author-provided mean, dispersion, and sample-size data. Thus, the phrase “fewer than three complete studies” means that the predefined threshold for formal pooling was not reached, not that biologically relevant methylation evidence was absent.

**Fig. 1:**
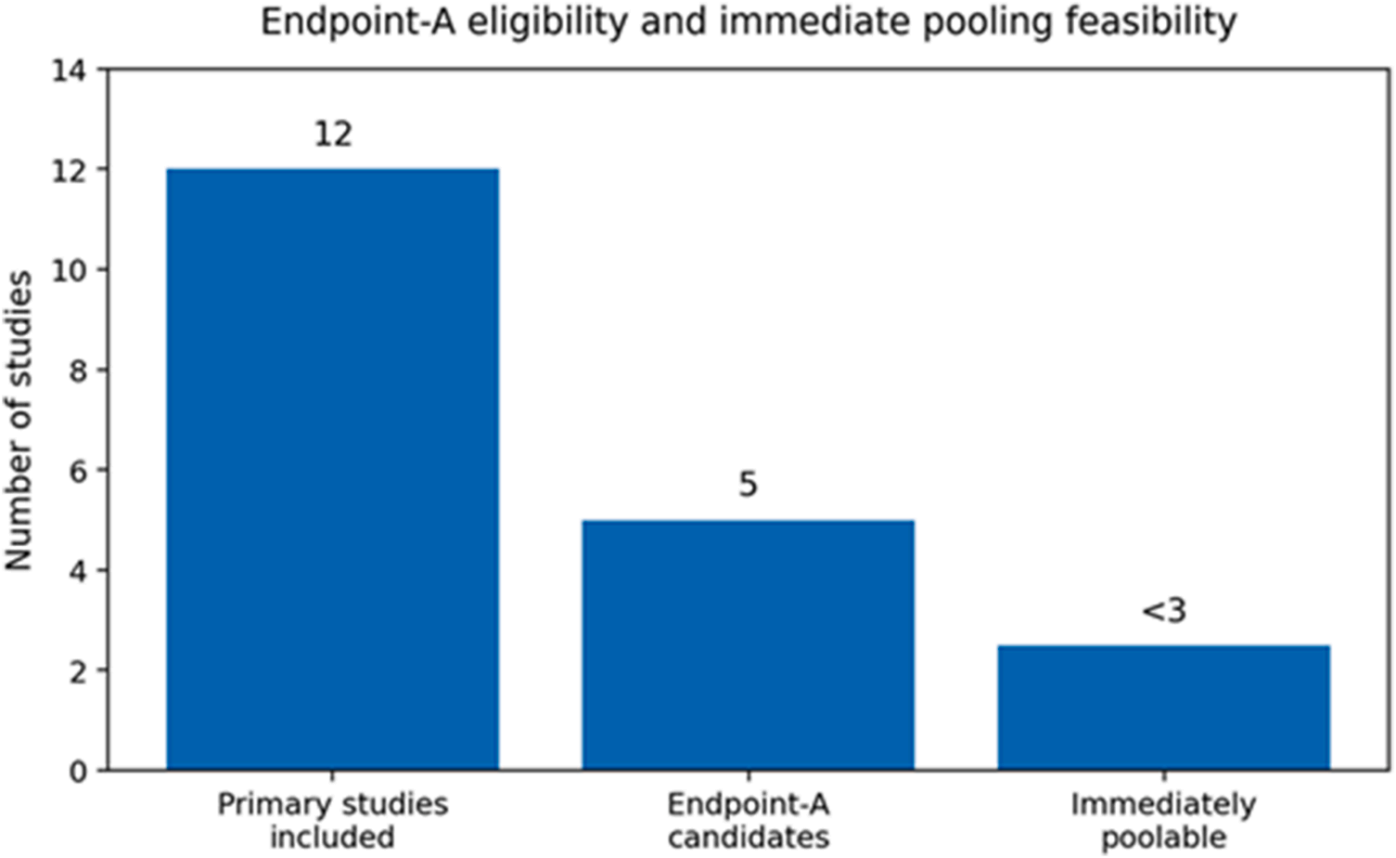
Endpoint-A eligibility and immediate pooling feasibility.

Fig. 1 visualizes the same attrition and makes clear that the barrier is endpoint comparability and reporting completeness, not the absence of biological evidence. Interpreted together, Table 2 and Fig. 1 show that the review question has two layers: a broad systematic-review layer that captures heat-induced methylome remodeling, and a narrow meta-analytic layer that only accepts comparable global methylation outcomes with complete group-level statistics.

The figure indicates that the field has moved rapidly toward high-resolution methylome mapping, while standard reporting of global methylation means, variances, and sample sizes has not kept pace.

### 3.2 Characteristics of included studies

Table 4 summarizes the principal studies included in the updated synthesis. The study designs are biologically complementary but statistically heterogeneous. Reproductive studies measured global methylation or hydroxymethylation in oocytes and embryos (Diaz et al., 2021; Diaz et al., 2025); immune-cell studies used PBMCs or blood mononuclear cells (Livernois et al., 2021; Aalam et al., 2022; Sajjanar et al., 2024); developmental-programming studies examined calves exposed prenatally or postnatally to heat (Laporta et al., 2025; Boucher et al., 2026); and multi-omics studies integrated methylation with transcriptomic or metabolic profiles (Halli et al., 2025). This diversity is scientifically valuable, but it also explains why unrestricted meta-analysis would be inappropriate.

**Table 4:** Study-level evidence extraction for bovine heat-stress methylation studies.

Material	Heat-stress model	Methylation endpoint	Main result	Role in synthesis	Study
GV and MII oocytes	Seasonal spring vs. summer heat stress	Global 5mC/5hmC; transcriptomics	No significant global methylation/hydroxymethylation difference; transcriptomic disruption and oocyte-quality effects.	Endpoint A eligible; limited extractable values.	Díaz et al., 2021
Blood mononuclear cells	Holstein cows with divergent immune-response phenotype under in vitro heat stress	Genome-wide promoter methylation	Differential promoter methylation related to stress response, apoptosis, proliferation and histone deacetylation.	Mechanistic; not poolable.	Livernois et al., 2021
Peripheral blood	Angus vs. Nellore under heat-stress adaptation context	Genome-wide methylome signatures	Breed-specific and shared methylation changes in genes and pathways related to thermal adaptation.	Mechanistic; not poolable.	del Corvo et al., 2021
Cultured PBMCs	Acute heat shock in zebu and crossbred dairy cattle	Global methylation plus targeted promoters	No significant global DNA methylation change; promoter-level changes in stress-related genes.	Endpoint A eligible, but numeric data incomplete.	Aalam et al., 2022
Oocytes and embryos	Control vs. heat stress during development	Methylation/hydroxymethylation signal intensity	Reduced epigenetic signal intensity and impaired developmental outcomes under heat stress.	Potential endpoint A; requires verified numeric extraction.	Feng et al., 2024
PBMC/methylome	Hariana vs. Vrindavani heat-stress response	Genome-wide CpG methylation and DMRs	Thousands of differentially methylated CpGs; integrated methylation-expression signals.	Mechanistic; not poolable.	Sajjanar et al., 2024
MII oocytes and early embryos	In vivo and in vitro heat stress	Global 5mC/5hmC immunostaining	Study directly tested methylation and hydroxymethylation in oocytes/embryos; public summaries suggest no simple universal effect.	Endpoint A candidate; numeric recovery needed.	Diaz et al., 2025
Liver biopsies from calves	Preweaning heat stress vs. cooling	RRBS DMCs plus RNA-seq	14,639 DMC mapping to 3,197 DMGs; pathways in PI3K/AKT, PPARGC1A, STAT5B, CREB and XBP1 regulation.	Mechanistic; not poolable.	Laporta et al., 2025
Blood of cows and female progeny	Direct and maternal heat stress	Methylation, transcriptomics, metabolomics	Multi-omics separation of heat-stressed vs. control groups; intergenerational molecular effects.	Mechanistic; not poolable.	Halli et al., 2025
Whole blood of dairy calves	Prenatal heat stress vs. prenatal cooling	Whole-genome enzymatic DNA methyl sequencing	682,898 DMC at birth and 97,289 persisting to weaning; pathways in immunity, inflammation and metabolism.	Mechanistic; not poolable as global endpoint.	Boucher et al., 2026

Table 4 shows substantial biological diversity across tissues, exposure models, and methylation platforms. This heterogeneity supports structured qualitative synthesis but precludes unrestricted quantitative pooling. The table, therefore, supports the core interpretation of the Results section: the biological signal is broad, but the statistical endpoint for meta-analysis must remain narrow. Oocytes and embryos mainly inform reproductive vulnerability, PBMC and whole-blood studies inform immune and systemic adaptation, and liver or prenatal-offspring studies inform metabolic and developmental programming. Because these biological matrices answer different questions, combining them into one pooled effect would obscure rather than clarify the evidence.

### 3.3 Meta-analysis feasibility: why a pooled estimate was not statistically defensible

The planned primary meta-analysis focused on global DNA methylation or hydroxymethylation. Applying this endpoint rule sharply reduced the quantitatively poolable evidence base. Several studies were biologically relevant but not statistically compatible because they reported DMC numbers, DMRs, promoter methylation, or pathway enrichment rather than a continuous global methylation value (Livernois et al., 2021; Del Corvo et al., 2021; Sajjanar et al., 2024; Laporta et al., 2025; Halli et al., 2025; Boucher et al., 2026). Among endpoint-A candidates, most did not provide all the group-level numerical information needed for SMD calculation in accessible text or summaries. Therefore, the final analysis is best described as a restricted meta-analytic assessment rather than a pooled meta-analysis, consistent with recommended practice when effect-size comparability and variance reporting are insufficient (DerSimonian and Laird, 1986; Higgins et al., 2003; IntHout et al., 2014; Balduzzi et al., 2019). A pooled estimate based on incompatible endpoints would be scientifically fragile. For example, a change in the number of DMCs is not equivalent to a change in mean 5mC intensity; a promoter-specific methylation shift cannot be interpreted as a whole-genome methylation shift; and methylation-transcriptome principal components represent multivariate separation rather than a single methylation effect size. The appropriate conclusion is that the current evidence supports methylome remodeling, but current reporting does not yet support a robust pooled estimate of global methylation response (Table 5).

**Table 5:** Restricted meta-analysis eligibility and pooling decision.

Endpoint-A eligibility	Quantitative status	Meta-analysis decision	Study or endpoint class
Yes: global 5mC/5hmC in oocytes	Partial values available; exact study-level data should be verified from the article/supplement before pooling.	Candidate, not sufficient alone.	Díaz et al., 2021
Yes: global DNA methylation in PBMCs	Publicly accessible summary confirms no significant global change, but not a complete extractable mean/SD/n in the available preview.	Candidate after full numeric extraction.	Aalam et al., 2022
Potentially yes: global-like methylation/hydroxymethylation signal intensity	The summary indicates reduced signal intensity, but complete numeric values require article-level extraction.	Candidate after verification.	Feng et al., 2024
Yes: global 5mC/5hmC in oocytes/embryos	Public summaries confirm endpoint; exact effect sizes require full text and supplementary data.	High-priority candidate.	Díaz et al., 2025
No for endpoint A	Biologically rich, but not a global continuous endpoint.	Narrative/mechanistic synthesis only.	Promoter methylation studies
No for endpoint A unless transformed into a prespecified comparable metric	Counts and pathway signatures are not equivalent to the mean methylation level.	Narrative/mechanistic synthesis only.	DMC/DMR/WGBS/RRBS studies

Table 5 is the key decision table for the restricted meta-analysis. It shows that endpoint-A eligibility is not the same as immediate poolability: several studies measure the correct biological construct but do not yet provide complete mean, variance, and sample-size information. Therefore, the correct conclusion is not that methylation evidence is weak, but that a numerical pooled estimate would currently be statistically fragile. This table justifies the statistical restraint adopted in the manuscript. Studies can be biologically important and still be excluded from the primary quantitative model if they report DMC counts, DMR enrichment, or promoter-specific methylation rather than a comparable continuous global methylation endpoint.

### 3.4 Extractable quantitative signal from global methylation studies

The clearest numerical values recoverable from the available materials were global methylation and hydroxymethylation signal summaries (Diaz et al., 2021). These values are useful for illustrating the endpoint-A framework, but they do not justify a pooled meta-analysis because they come from one evidence stream and require verification of denominators and variance structure before formal effect-size calculation. Directionally, they show no statistically significant seasonal difference in global methylation or hydroxymethylation at GV or MII stages, despite evidence of heat-stress-associated transcriptomic disturbance (Diaz et al., 2021; Roth, 2020; Hansen, 2009). Table 6 provides the clearest extractable numerical illustration of endpoint A. The pattern is biologically informative but not statistically definitive: GV-stage values are numerically lower during hot months, whereas MII-stage values do not follow the same direction.

**Table 6:** Recoverable global methylation values illustrating endpoint-A quantitative extraction.

Stage	Marker	Cool months, mean ± SE	Hot months, mean ± SE	Interpretation
GV oocytes	DNA methylation	417,218.90 ± 71,793.86	313,819.88 ± 55,528.01	Lower numerical signal in hot months, but reported as non-significant.
GV oocytes	DNA hydroxymethylation	444,931.10 ± 67,920.78	352,254.68 ± 56,425.96	Lower numerical signal in hot months, but reported as non-significant.
MII oocytes	DNA methylation	87,122.36 ± 14,449.47	89,807.26 ± 11,303.72	No meaningful directional decline.
MII oocytes	DNA hydroxymethylation	102,933.83 ± 15,517.70	137,622.45 ± 11,826.86	Higher numerical signal in hot months, but reported as non-significant.

**Fig. 2:**
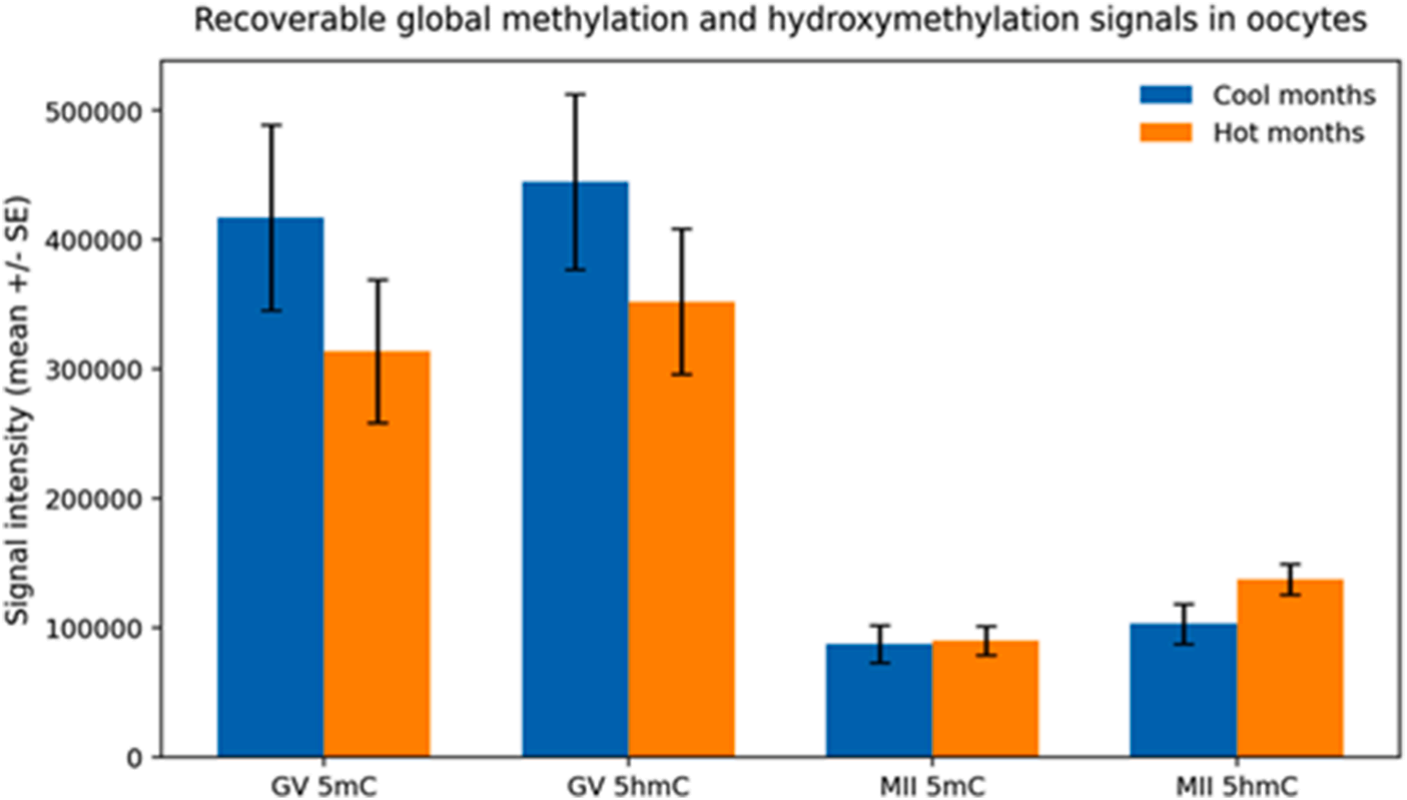
Recoverable global methylation and hydroxymethylation values from the oocyte evidence stream. Error bars show standard errors.

Fig. 2 presents these values graphically and reinforces the interpretation that heat stress should not be described as causing a simple global hypermethylation or hypomethylation response. According to Table 5, the values suggest that heat exposure may modify methylation signals in an oocyte-stage-dependent manner, but the overlapping uncertainty and incomplete replication prevent a claim of a consistent global methylation effect.

### 3.5 Locus-specific methylome remodeling

The major positive finding of this review is not a consistent global methylation shift, but extensive locus-specific remodeling. Blood and PBMC studies showed promoter-level or CpG-level differences associated with immune function, stress signaling, and breed-dependent thermotolerance (Livernois et al., 2021; del Corvo et al., 2021; Aalam et al., 2022; Sajjanar et al., 2024). Liver studies in calves exposed to postnatal heat stress revealed methylation changes linked to metabolic signaling and cellular homeostasis (Laporta et al., 2025). Available prenatal heat-stress studies suggest a large burden of differentially methylated cytosines at birth, with evidence that a subset of these marks may persist into early life, although cross-study replication remains limited (Boucher et al., 2026). Multi-omics work further supports the same interpretation by showing coordinated methylation, transcriptomic, and metabolic separation between heat-stressed and control groups (Halli et al., 2025). These findings support the view that cattle respond to heat stress through targeted regulatory remodeling rather than genome-wide methylation collapse.

Table 7 shifts the Results section from statistical eligibility to biological convergence. Across tissues and developmental windows, methylation signals repeatedly map to stress response, immunity, metabolism, reproductive competence, and developmental programming. Table 6, therefore, provides the strongest mechanistic evidence supporting the article title: heat stress appears to reprogram bovine methylomes in a targeted, locus-specific manner. The table also explains why the article remains scientifically strong despite the restricted meta-analysis: repeated convergence on stress, immune, metabolic, reproductive, and developmental pathways provides mechanistic consistency across otherwise heterogeneous platforms.

**Table 7:** Mechanistic convergence across locus-specific methylome studies.

Biological domain	Repeated methylation signal	Interpretation for heat-stress biology	Representative studies
Reproductive cells	Global methylation often stable, but oocyte/embryo competence and transcriptomic profiles altered.	Heat stress may affect reproductive competence through targeted transcriptional and cellular mechanisms without requiring a large global methylation shift.	Diaz et al., 2021; Diaz et al., 2025; Feng et al., 2024.
Immune cells and PBMCs	Promoter and CpG methylation differences in stress-responsive and immune pathways.	Methylation may contribute to immune phenotype, inflammation, cell survival, and thermotolerance variation.	Livernois et al., 2021; Aalam et al., 2022; Sajjanar et al., 2024.
Breed adaptation	Angus, Nellore, Zebu, and crossbred comparisons show genotype-dependent methylation patterns.	Thermotolerance may involve both genetic background and environmentally responsive methylome regulation.	del Corvo et al., 2021; Sajjanar et al., 2024.
Prenatal programming	Large DMC burden at birth following prenatal heat stress, with partial persistence to weaning.	The prenatal thermal environment may leave measurable blood methylation signatures relevant to immunity, inflammation and metabolism.	Boucher et al., 2026; Halli et al., 2025.
Postnatal calf liver	DMCs and DMGs in signaling and metabolic pathways after preweaning heat stress.	Early-life heat exposure may alter hepatic regulatory pathways linked to growth, metabolism, and cellular stress.	Laporta et al., 2025.

### 3.6 Risk-of-bias and reporting quality

The overall risk-of-bias profile was moderate. Most studies clearly defined biological material and a methylation platform, but fewer reported complete group-level methylation values suitable for meta-analysis. Several sequencing studies provided strong mechanistic data but were not designed for pooled global methylation analysis. Common limitations included small sample size, heterogeneous heat-stress thresholds, limited adjustment for cell-type composition, incomplete reporting of variance estimates, and inconsistent integration of methylation with physiological outcomes (Table 8). These issues are common in emerging epigenomic evidence syntheses, where tissue specificity, platform choice, and cell-composition effects strongly influence comparability (Rakyan et al., 2011; Pidsley et al., 2016; Wang et al., 2021).

**Table 8:** Study-level risk-of-bias and reporting-quality judgments for included bovine heat-stress methylation studies.

Study	Heat-stress definition and comparator	Replication/confounding control	Assay/reporting quality	Overall judgment
Díaz et al., 2021	Clear seasonal comparison; comparator appropriate.	Moderate; reproductive material and season introduce biological variability.	Global 5mC/5hmC values partially extractable; variance structure needs verification.	Moderate
Livernois et al., 2021	Clear in vitro heat-stress/immune-phenotype context.	Moderate; phenotype groups defined but cell-composition effects remain relevant.	Strong promoter-methylation assay; not a global endpoint.	Moderate
Del Corvo et al., 2021	Breed/adaptation context clear; comparator biologically meaningful.	Moderate; breed and environment may be partly confounded.	Genome-wide methylome data informative; not endpoint A.	Moderate
Aalam et al., 2022	Acute heat-shock model clear.	Moderate; zebu/crossbred comparison informative but sample details limit pooling.	Global and promoter endpoints reported; group-level numerical extraction incomplete.	Moderate
Feng et al., 2024	Oocyte heat-stress model clear.	Moderate; developmental-stage effects important.	Global-like signal intensity relevant but requires verified complete numerical extraction.	Moderate
Sajjanar et al., 2024	Heat-stress response in Hariana/Vrindavani clear.	Moderate; breed and physiological background require cautious interpretation.	Genome-wide CpG/DMR evidence strong; not a global continuous endpoint.	Moderate
Díaz et al., 2025	In vivo/in vitro heat-stress exposure clear.	Moderate; reproductive stage and embryo/oocyte context.	Endpoint-A candidate; exact effect-size extraction requires full data verification.	Moderate
Laporta et al., 2025	Preweaning heat stress vs cooling clear.	Moderate to low risk; controlled calf study context.	RRBS/RNA-seq strong mechanistic reporting; not endpoint A.	Low-moderate
Halli et al., 2025	Direct and maternal heat-stress contrasts clear.	Moderate; multi-generational design complex.	Integrated methylation/transcriptomic/metabolic evidence; not global endpoint.	Moderate
Boucher et al., 2026	Prenatal heat stress vs cooling clear.	Moderate to low risk; longitudinal calf sampling strengthens inference.	Whole-genome enzymatic methyl sequencing; not poolable as global endpoint.	Low-moderate

## 4 Discussion

### 4.1 Biological interpretation, translational relevance, and meta-analytic limits

#### 4.1.1 Principal findings

This systematic review shows that heat stress is associated with meaningful epigenomic alteration in cattle, but the alteration is not best described as a universal increase or decrease in global methylation. The strongest interpretation is that bovine heat stress induces tissue-specific, exposure-specific, and developmental-stage-specific methylome remodeling. This remodeling is most visible in sequencing-based studies that detect DMCs, DMRs, promoter signatures, and integrated methylation-transcriptome or methylation-metabolome patterns (Livernois et al., 2021; del Corvo et al., 2021; Sajjanar et al., 2024; Laporta et al., 2025; Halli et al., 2025; Boucher et al., 2026). This conclusion is also biologically coherent with the broader heat-stress literature showing that thermal load affects reproduction, welfare, immunity, endocrine status, and cellular homeostasis in cattle (Polsky and von Keyserlingk, 2017; Roth, 2020; Rhoads, 2023; Lacetera, 2019).

The restricted meta-analysis question produced a different conclusion. For the predefined primary endpoint of global methylation or hydroxymethylation, the literature remains too sparse and incompletely reported for a robust pooled estimate. This is not a failure of the review; it is an important scientific result. It tells the field that current evidence supports the existence of methylation responses, but not yet the estimation of a single pooled global effect size. Tables 3 to 6 and Figs. 1 to 3 together show this logic: the search identified enough studies for systematic synthesis, but endpoint-A attrition and incomplete reporting prevent a defensible pooled estimate. This interpretation follows established meta-analytic principles: when studies do not measure the same construct or do not report extractable means, dispersion estimates, and sample sizes, a pooled summary can be more misleading than informative (DerSimonian and Laird, 1986; Higgins et al., 2003; IntHout et al., 2014; Balduzzi et al., 2019).

#### 4.1.2 Why global and locus-specific methylation lead to different messages

Global methylation assays average signal across large genomic compartments. They are useful for detecting broad epigenomic shifts, but they may miss targeted changes at stress-responsive genes, regulatory regions, or tissue-specific enhancers. Under heat stress, cattle likely activate precise regulatory responses involving heat-shock proteins, oxidative-defense pathways, glucocorticoid signaling, metabolic rewiring, immune modulation, and cell-survival programs (Aalam et al., 2022; Livernois et al., 2021; Sajjanar et al., 2024). Such responses can generate thousands of local methylation differences without changing the overall methylation mean enough to be detected by bulk assays, a pattern consistent with broader epigenome-wide association principles and platform-dependent methylation coverage (Rakyan et al., 2011; Pidsley et al., 2016).

This explains why global methylation studies can be statistically null while genome-wide studies show extensive remodeling. A null global result should not be interpreted as the absence of epigenetic response; it means that the response may be spatially specific within the genome (Diaz et al., 2021; Aalam et al., 2022). Conversely, a large DMC burden should not be interpreted as a global methylation shift unless a global metric is directly measured (del Corvo et al., 2021; Sajjanar et al., 2024; Laporta et al., 2025; Boucher et al., 2026). Table 5, Table 6 make this distinction visible: the studies closest to quantitative pooling do not show a uniform global direction, while sequencing studies outside endpoint A provide the strongest mechanistic signal.

#### 4.1.3 Biological relevance of heat-stress methylome remodeling

The biological convergence across studies is strong and is summarized in Table 6. Reproductive studies link heat exposure to oocyte and embryo vulnerability (Diaz et al., 2021; Diaz et al., 2025; Hansen, 2009; Roth, 2020). Immune-cell studies link methylation changes to stress signaling and immune phenotype (Livernois et al., 2021; Aalam et al., 2022; Sajjanar et al., 2024). Breed-comparison studies suggest that thermotolerant genotypes may differ not only genetically but also epigenetically (Del Corvo et al., 2021; Sajjanar et al., 2024). Prenatal and postnatal calf studies show that thermal exposure during sensitive developmental windows can leave methylation signatures in blood or liver (Laporta et al., 2025; Halli et al., 2025; Boucher et al., 2026). Together, these findings support the concept of climate-induced epigenetic reprogramming in cattle.

In this sense, it is confirmed that heat stress affects pathways that are directly relevant to cattle productivity and welfare, including oocyte competence, immune regulation, oxidative balance, energy metabolism, and developmental programming. These pathway-level findings are more useful for biological interpretation than a single pooled global methylation estimate would be at the current stage of the literature.

The most translationally relevant pathways include oxidative stress, inflammatory regulation, cellular signaling, energy metabolism, glucocorticoid signaling, mitochondrial function, and developmental control. These pathways are consistent with the known physiology of heat stress: reduced feed intake, altered endocrine status, inflammatory pressure, impaired fertility, changes in immune competence, and altered nutrient partitioning (Polsky and Von Keyserlingk, 2017; Lacetera, 2019; Roth, 2020; Rhoads, 2023). Methylation is unlikely to be the only mechanism involved; histone modifications, chromatin accessibility, non-coding RNAs, and microbiome-related metabolites may also participate (Wang et al., 2021; Halli et al., 2025). However, methylation has particular value because it can be measured in accessible tissues and may serve as a biomarker of exposure or resilience if validated against phenotypes such as fertility, immune competence, calf growth, thermoregulation, and survival.

The broader heat-stress and oxidative-stress literature supports this mechanistic framing. Work on epigenetic regulation of cellular stress responses and antioxidant defense indicates that oxidative stress can activate protective redox pathways and apoptosis-related responses, while occupational heat-exposure studies show that cooling garments and heat-management interventions can reduce physiological strain under high-temperature conditions (Mishra et al., 2022; Tripathi et al., 2025; Ashtekar et al., 2019; Shirish et al., 2016). These studies are not bovine DNA-methylation studies and were therefore not counted as primary evidence in the systematic synthesis; however, they strengthen the biological plausibility that thermal load, oxidative imbalance, epigenetic regulation, and protective cooling interventions are connected across mammalian systems and applied heat-exposure settings.

#### 4.1.4 Implications for cattle breeding and management

For animal breeding, methylation signatures may eventually help identify animals that maintain cellular homeostasis under heat. This is particularly relevant because cattle breeds and genotypes differ in thermotolerance and in heat-stress-associated methylome patterns (Del Corvo et al., 2021; Sajjanar et al., 2024). However, epigenetic biomarkers should not be used prematurely. A biomarker must be reproducible across cohorts, tissues, breeds, ages, and laboratories, and it must predict a meaningful phenotype such as fertility, immune competence, survival, growth, or milk-production stability under heat (Rakyan et al., 2011; Wang et al., 2021). Current studies provide candidate regions and pathways, not yet validated selection tools.

For farm management, the strongest practical implication remains prevention and mitigation of heat stress. Epigenetic evidence should not be used to justify exposing animals to heat in the hope of inducing adaptation. Cooling, shade, ventilation, water access, nutritional support, and welfare protection remain essential because climate-related heat load directly affects animal welfare, health, and productivity (Polsky and von Keyserlingk, 2017; Lacetera, 2019; Rojas-Downing et al., 2017). The methylation literature strengthens the argument for protecting pregnant cows, oocytes, embryos, and young calves because early-life thermal exposure may leave persistent molecular marks and later-life phenotypic consequences (Hansen, 2009; Roth, 2020; Skibiel et al., 2021; Laporta et al., 2025; Boucher et al., 2026).

### 4.2 Limitations and recommendations for future studies

This review has three main limitations. First, no prospective protocol was registered, and the review should therefore be presented as PRISMA-informed rather than as a fully preregistered systematic review. Second, the evidence base remains heterogeneous in tissue type, breed background, heat-stress definition, exposure duration, methylation platform, and endpoint reporting. Third, several biologically important studies could not contribute to a pooled global effect because they reported DMCs, DMRs, promoter-level signatures, pathway enrichment, or multivariate omics separation rather than complete group-level global 5mC/5hmC statistics.

For future work, the highest priority is not simply to increase sample size but to improve comparability and reporting. Studies should report THI or equivalent heat-load metrics, exposure duration, cooling conditions, breed and parity, developmental stage, tissue processing, cell-composition assessment, methylation platform and quality-control parameters, group-level means with standard deviations or standard errors, exact sample sizes, and links between methylation signatures and phenotypes. These elements would permit rigorous subgroup analysis, meta-regression, and ultimately the validation of methylation-based indicators of heat resilience.

## 5 Conclusion

Heat stress is associated with measurable DNA methylation remodeling in cattle, but the available evidence does not support a simple universal model of global hypermethylation or hypomethylation. The most defensible interpretation is that heat stress induces tissue-specific, locus-specific, and developmental-stage-specific methylome remodeling across reproductive cells, embryos, PBMCs, whole blood, liver, and prenatally exposed calves. These methylation signals converge on biological pathways involved in oxidative stress and immune regulation, metabolism, endocrine signaling, cellular homeostasis, and developmental programming.

A formal pooled meta-analysis of global DNA methylation or hydroxymethylation is not currently defensible because fewer than three independent studies provide complete and comparable group-level data for the prespecified continuous endpoint. This limitation does not negate the biological evidence for heat-stress-associated epigenomic remodeling; rather, it identifies the principal reporting barrier preventing quantitative synthesis. Future bovine heat-stress epigenomics studies should provide standardized heat-load metrics, precise tissue and cell-composition information, extractable means and dispersion estimates, open methylation matrices, and phenotype-linked outcomes for fertility, immunity, production, growth, thermoregulation, and welfare. With these improvements, the field can move from candidate methylation signatures toward validated biomarkers and mechanistic models of climate resilience in cattle.

## Statement for studies in humans/animals

Not applicable, as this review did not involve the use of animals.

## Declarations

### Ethics approval and consent to participate

Not applicable; this article is a review of previously published studies.

### Declaration of generative AI and AI-assisted technologies in the manuscript preparation process

The authors used ChatGPT (OpenAI) to improve language and structure. They reviewed and edited the manuscript and take full responsibility for the final content.

### Data availability

All data summarized in this review were extracted from published articles and public article records. No new animal or laboratory data were generated.

### Funding

This research received no specific grant from any funding agency in the public, commercial, or not-for-profit sectors.

## CRediT authorship contribution statement

**Safa Bejaoui:** Writing – original draft, Data curation. **Ikram Bensouf:** Writing – original draft, Data curation. **Lotfi Mhamdi:** Writing – review & editing. **Naceur M'Hamdi:** Writing – review & editing, Validation, Supervision, Resources, Conceptualization.

## Declaration of competing interest

The authors declare that they have no known competing financial interests or personal relationships that could have appeared to influence the work reported in this paper.
